# *Mycobacterium bovis* BCG increase the selected determinants of monocyte/macrophage activity, which were diminished in response to gastric pathogen *Helicobacter pylori*

**DOI:** 10.1038/s41598-023-30250-6

**Published:** 2023-02-22

**Authors:** Weronika Gonciarz, Maciej Chyb, Magdalena Chmiela

**Affiliations:** 1 Department of Immunology and Infectious Biology, Faculty of Biology and Environmental Protection, University of Lodz, 12/16 Banacha St., 90-237 Lodz, Poland; 2 Department of Molecular Microbiology, Faculty of Biology and Environmental Protection, University of Lodz, Lodz, Poland; 3 Bio-Med-Chem Doctoral School of the University of Lodz and Lodz Institutes of the Polish Academy of Sciences, Lodz, Poland

**Keywords:** Monocytes and macrophages, Bacterial host response

## Abstract

High antibiotic resistance of gastric pathogen *Helicobacter pylori* (Hp) and the ability to escape the host immune response prompt searching for therapeutic immunomodulators. Bacillus Calmette–Guerin (BCG) vaccine with *Mycobacterium bovis* (Mb) is a candidate for modulation the activity of immunocompetent cells, and onco-BCG formulation was successfully used in immunotherapy of bladder cancer. We determined the influence of onco-BCG on the phagocytic capacity of human THP-1 monocyte/macrophage cells, using the model of *Escherichia coli* bioparticles and Hp fluorescently labeled. Deposition of cell integrins CD11b, CD11d, CD18, membrane/soluble lipopolysaccharide (LPS) receptors, CD14 and sCD14, respectively, and the production of macrophage chemotactic protein (MCP)-1 were determined. Furthermore, a global DNA methylation, was also assessed. Human THP-1 monocytes/macrophages (TIB 202) primed or primed and restimulated with onco-BCG or Hp, were used for assessment of phagocytosis towards *E. coli* or Hp, surface (immunostaining) or soluble activity determinants, and global DNA methylation (ELISA). THP-1 monocytes/macrophages primed/restimulated with BCG showed increased phagocytosis capacity towards *E. coli* fluorescent particles, elevated expression of CD11b, CD11d, CD18, CD14, sCD14, increased MCP-1 secretion and DNA methylation. Preliminary results indicate that BCG mycobacteria may also induce the phagocytosis of *H. pylori* by THP-1 monocytes. Priming or priming and restimulation of monocytes/macrophages with BCG resulted in an increased activity of these cells, which was negatively modulated by Hp.

## Introduction

*Helicobacter pylori,* spiral Gram-negative rods, colonize the gastric mucosa in humans (on average 50% population), and induce the development of chronic inflammatory response, gastric or duodenal ulcers, and gastric cancer^1–3^. The role of *H. pylori* in the development of extragastric diseases has been also suggested^4–6^. Locally in the stomach, *H. pylori* disintegrate gastric epithelial barrier due to elevated oxidative stress and an increased cell apoptosis^7,8^. World Health Organization (WHO) reported that resistance of *H. pylori* to antibiotics, including amoxicillin, clarithromycin, metronidazole, levofloxacin, used for treatment of infection is growing^9–12^. High antibiotic resistance together with different strategies of *H. pylori* to avoid the host immune responses may influence the effectiveness of eradication. Components of *H. pylori* diminish phagocytic activity of phagocytes^13–16^, and expansion as well as cytotoxic activity of natural killer cells (NK)^17^. Moreover, proliferation of T lymphocytes is affected in response to secreted or cell bound *H. pylori* compounds, while those which share common sequences with the host proteins may induce antibodies, potentially autoreactive^6,8,18–20^. The idea of using immunomodulating formulations for supporting the treatment of *H. pylori* is taken into account. Immunostimulants modulate the activity of innate immune cells, including monocytes and NK cells. Potentially it may result in an increased activity against homologous or heterologous infectious agents^20–24^. Increased anti-microbial activity of “trained cells” might be achieved through epigenetic modifications in genes encoding the immune mediators or by metabolic reprogramming of cells by microbial components^25,26^. *H. pylori* influence the first line of defense, where monocytic-macrophage cells play a key role. This prompts the search for formulations restoring the activity of these cells. Macrophages eliminate infectious agents as well as apoptotic cells by phagocytosis, and recover local homeostasis. *Mycobacterium bovis* Bacillus Calmette–Guerin (BCG) vaccine strain has been considered as candidate immunomodulator for macrophages^27,28^. It has been suggested that BCG may induce non-specific immune memory in innate immune cells that may be cross-protective against various not related pathogens. Several studies revealed, that BCG vaccination may reduce mortality of infants infected with different infectious agents and induce a better innate immune response against microorganisms other than *Mycobacterium tuberculosis*, including *Candida albicans, Staphylococcus aureus,* respiratory syncytial virus, and potentially Sars-Cov-2^29–31^.

Onco-BCG vaccine has developed into the most mature immunotherapy for bladder cancer^32^. The potential mechanisms involve binding of BCG to tumor cell fibronectin and internalization of mycobacteria by tumor cells. Through variety of intracellular signal transduction pathways, cell apoptosis, cell necrosis and an oxidative stress BCG induce tumor cell death. On the other hand, mycobacteria induce cytokines, which drive an immune cascade that facilitates the host’s immune system to kill tumor cells^32^.

The aim of this study was to determine whether *M. bovis* onco-BCG vaccine formulation is able to improve phagocytic capacity of human THP-1 monocytes, which was affected in cell cultures in vitro by *H. pylori*. Furthermore, whether the exposure of THP-1 derived macrophages to BCG mycobacteria will influence the expression of surface integrins CD11b, CD11d, CD18, membrane and soluble lipopolysaccharide (LPS) receptors, CD14 and sCD14, respectively, and the production of macrophage chemotactic protein (MCP)-1. The global DNA methylation will be assessed as preliminary marker of epigenetic modifications.

## Results and discussion

### Upregulation by *M. bovis* BCG of monocyte/macrophage phagocytic capacity diminished in response to *H. pylori*

**Figure 1:**
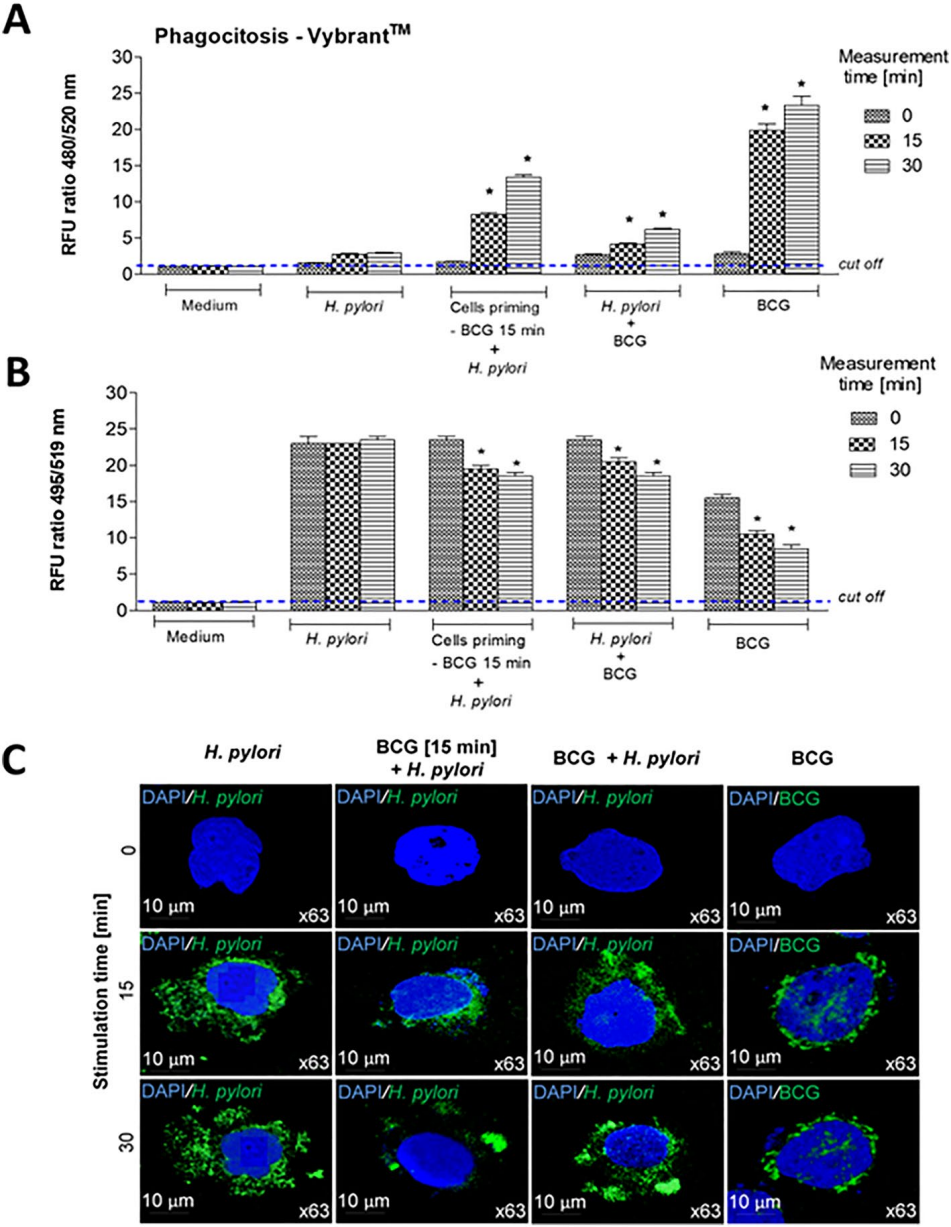
The influence of short exposure of THP-1 monocytes to *M. bovis* BCG on their phagocytic properties towards fluorescently labeled *E. coli* particles or *H. pylori*. The ingestion by THP-1 cells of fluorescently labeled *E. coli* (Vybrant phagocytosis assay kit) (**A**) was assessed in time 0 and after 15 min and 30 min using the following variants of cell cultures: cells exposed to *H. pylori* alone, cells exposed to BCG (15 min) and then to *H. pylori*, cells exposed simultaneously to BCG and *H. pylori*, or cells exposed to BCG alone or cells in RPMI-1640 medium alone (control). The fluorescence of intracellular bacteria was measured. The phagocytic capacity of THP-1 cells towards fluorescently labeled *H. pylori* (**B**) was assessed in time 0 and after 15 min and 30 min using the following variants of cell cultures: cells exposed to fluorescently labeled *H. pylori* alone, cells exposed to unlabeled BCG (15 min) and then to fluorescently labeled *H. pylori*, cells exposed simultaneously to unlabeled BCG and fluorescently labeled *H. pylori*, or cells exposed to fluorescently labeled BCG alone. The intensity of fluorescently labeled bacteria remaining in the supernatants was measured. Results (**A**, **B**) are presented as ratio of median fluorescence units (RFU) ± range of three independent experiments. The difference statistically significant when *p* < 0.05 in Mann–Whitney *U* test. *Cells stimulated versus unstimulated (according to the time of stimulation). Images of THP-1 cells exposed to fluorescently labeled *H. pylori* or *M. bovis* BCG alone, to unlabeled *M. bovis* BCG and then to fluorescently labeled *H. pylori*, or simultaneously to unlabeled *M. bovis* BCG and fluorescently labeled *H. pylori*, in the fluorescence microscope (**C**). BCG or *H. pylori* MOI 10:1.

*H. pylori* developed several mechanism of escaping the immune mechanisms of the host, including the effectiveness of phagocytosis^13–15^. It has been suggested that an enhanced infiltration of macrophages to the gastric mucosa colonized by *H. pylori* may increase the elimination of these bacteria directly due to phagocytosis or due to induction of specific immune responses in the gut lymphoid tissue. It might also help to eliminate cells undergoing apoptosis, the number of which is increased during infection with *H. pylori*^7,33^. In this study we used in vitro model of THP-1 monocyte cell line to assess whether *M. bovis* BCG may upregulate the global phagocytic capacity of these cells, which was diminished by *H. pylori*, by measurement the ability of phagocytes to engulf fluorescently labeled *E. coli* bioparticles included in Vybrant phagocytosis assay, which is a model system for quantitating the effects of different factors on phagocytic function. This technique takes advantage of the detectability of the intracellular fluorescence emitted by the engulfed particles, as well as the effective fluorescence quenching of the extracellular probe by trypan blue. Phagocytic capacity of monocytes was assessed using cells exposed for 15 or 30 min to *H. pylori* or cells exposed for 15 or 30 min to BCG alone, to compare whether phagocytes are able to ingest *H. pylori* or BCG onco. Furthermore, monocytes primed with BCG for 15 min and then exposed to *H. pylori* for 15 or 30 min were used to determine whether short exposure of phagocytes to BCG onco will influence the phagocytic capacity of monocytes. The exposure time was selected experimentally in the preliminary study. The monocytes, which were exposed simultaneously to *H. pylori* and BCG for 15 or 30 min consisted an additional experimental variant (Fig. 1). We estimated the phagocytic capacity of such variants of THP-1 monocytes towards fluorescently labeled *E. coli* by measurement the fluorescence of cells with engulfed bacteria (localized intracellularly) (Fig. 1A).

Cells exposed to BCG showed an increased phagocytic capacity towards *E. coli* as compared to control cells in RPMI-1640 medium alone. By comparison, phagocytic capacity of THP-1 cells exposed to *H. pylori* did not increase *vs* control cells (Fig. 1A). However, priming of THP-1 cells for 15 min with BCG and then the exposure of them to *H. pylori* or simultaneously to BCG and *H. pylori* resulted in enhanced phagocytosis of fluorescently labeled *E. coli* as compared to cells treated with *H. pylori* alone (Fig. 1A). The effectiveness of phagocytosis of *E. coli* by cells primed with BCG and then with *H. pylori* was better than phagocytic activity of cells exposed to BCG and *H. pylori* mix. These results show that short priming of monocytes with BCG-onco improved their phagocytic capacity, which was diminished by *H. pylori*.

Furthermore we assessed the phagocytic capacity of THP-1 monocytes specifically towards *H. pylori* or BCG alone, labelled fluorescently using commercial LIVE/DEAD BacLight. In this experimental variant we assessed the fluorescence of bacteria remaining in the cell culture supernatants after co-incubation of bacteria with the phagocytes (Fig. 1B). We showed that BCG bacilli were effectively engulfed by phagocytes after 15 min or 30 min of exposure while phagocytosis of *H. pylori* was not enhanced. The lower RFU ratio in BCG alone at 0 min, which is different from the *H. pylori* variant alone, might be the effect of different sensitivity of *H. pylori* vs BCG bacteria for fluorescence staining procedure, but not MOI, which was the same (10:1). However, this difference did not affect the analyzed results, which showed that phagocytes were able to ingest BCG bacilli, while *H. pylori* were not ingested. Furthermore, we used THP-1 monocytes primed for 15 min with BCG, which were not labeled. Then monocytes were exposed for 15 min or 30 min to fluorescently labelled *H. pylori.* We showed that such cells were activated to phagocytize *H. pylori* as compared to cells exposed to *H. pylori* alone. Similarly, cells treated simultaneously with mix of unlabeled BCG and labeled *H. pylori* showed better phagocytic capacity towards *H. pylori* than cells not treated with BCG (Fig. 1B). These preliminary results indicate that the phagocytic activity of monocytes was induced by BCG onco and resulted in ingestion of *H. pylori*.

We also looked for the cell images under fluorescent microscope. We could see that monocytes exposed to *H. pylori* alone after 15 min and 30 min were mainly coated with the clumps of these bacteria. Only some patches with green fluorescence were observed in the nuclear regions, which may indicate the intracellular localization of some *H. pylori* bacteria (Fig. 1C). In the case of cells treated with BCG alone we could see an intracellular localization of mycobacteria. Furthermore, in the images of monocytes primed with BCG and then exposed to *H. pylori* the clumps of *H. pylori* were smaller than those surrounding the phagocytes primed with *H. pylori* alone. Similar, although weaker effect was showed for phagocytes simultaneously treated with BCG and *H. pylori* (Fig. 1C). These preliminary results indicate that the priming of THP-1 monocytes with BCG for 15 min or the presence of BCG in the mix suspension with *H. pylori* which was added to phagocytes resulted in an increased uptake of *H. pylori*. Potentially it might be due to very effective adhesive properties of mycobacteria, which use phagocytes as target cells^34,35^. The fluorescence of supernatants in these experimental variants was significantly decreased after 15 min and 30 min of phagocytosis procedure with *H. pylori* as compared to 0 min time point. However, the measurements of RFU ratio after 15 min and 30 min were comparable, which indicate that the intensity of phagocytosis in these two time points was similar and meaning that maximum of engulfment was reached between 15 and 30 min. In our model of *H. pylori* phagocytosis, which was examined using LIVE/DEAD BacLight staining procedure it is unclear why if *H. pylori* formed big clumps around the THP-1 cell surface, the fluorescence of bacteria remaining in the culture supernatants in 15 min or 30 min in this group was not decreased compared to the 0 min time in this group. It could be due to release of fluorescence from *H. pylori* bacterial clumps or possible from some bacteria undergoing degradation. It may be also possible that soluble components of *H. pylori* i.e. urease or mediators delivered by macrophages tightly coated with labeled bacteria may drive the increased fluorochrome releasing. These results indicate that *H. pylori* phagocytosis is a complex process, which might be influenced by different components in the experimental milieu and prompt deeper analysis of *H. pylori* interaction with macrophages. Further studies are needed to elucidate the exact mechanism induced by BCG mycobacteria in elevation of phagocytic capacity of monocytes. Particularly, this effect is interesting in terms of enhancement of phagocytosis of *H. pylori*, which can modulate negatively this process^13–16^.

**Figure 2:**
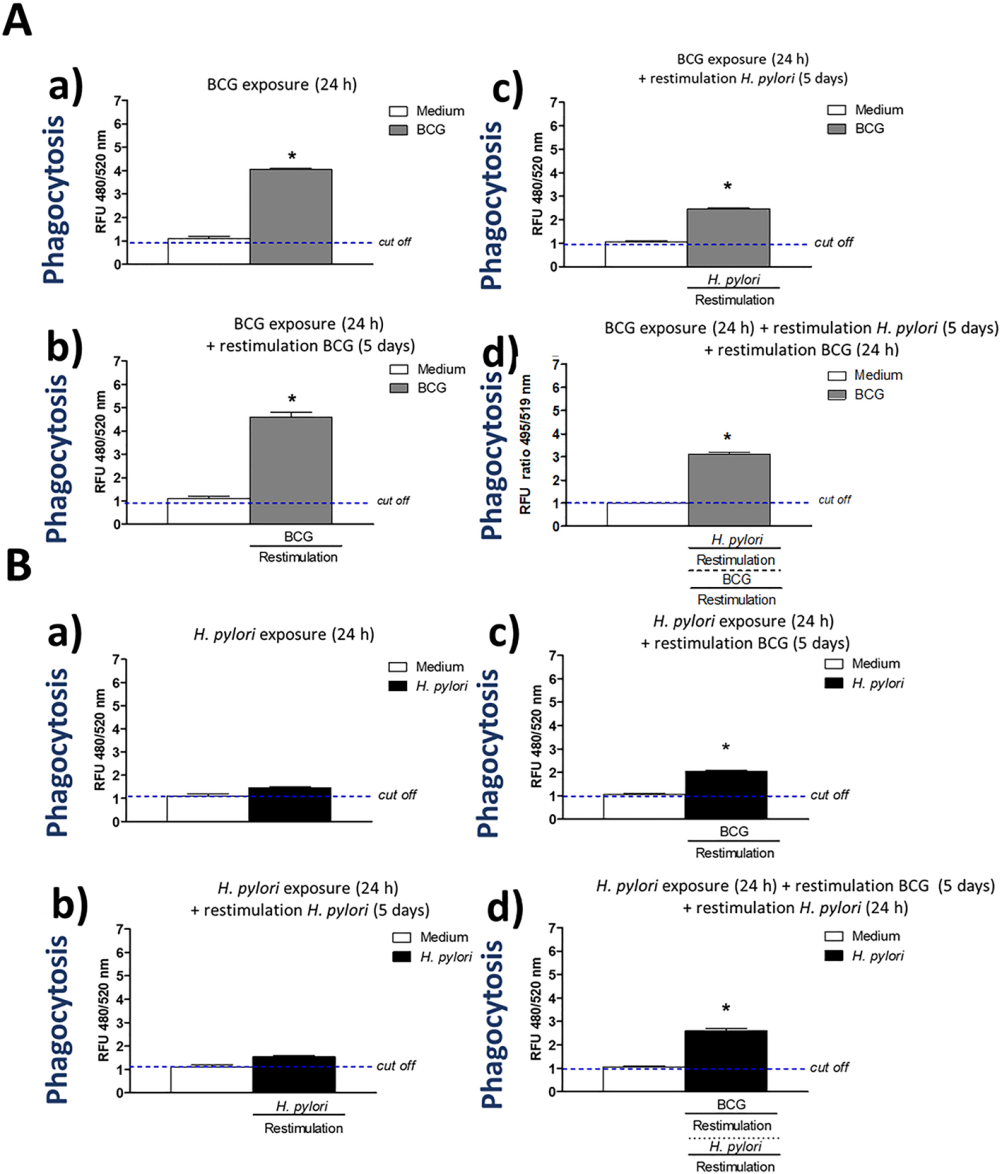
The role of priming/restimulation of THP-1 macrophages with *M. bovis* BCG in the enhancement and the maintenance of increased phagocytic activity of these cells in Vybrant phagocytosis assay using green-labeled *E. coli*. Phagocytic activity of THP-1 macrophages primed with *M. bovis* BCG (**A**) towards *E. coli*: cells primed for 24 h with *M. bovis* BCG (a); cells primed for 24 h with *M. bovis* BCG and restimulated for 5 days with *M. bovis* BCG (b); cells primed for 24 h with *M. bovis* BCG and restimulated for 5 days with *H. pylori* (c); cells primed for 24 h with *M. bovis* BCG then restimulated for 5 days with *H. pylori* and for an additional 24 h with *M. bovis* BCG (d). Phagocytic activity of THP-1 derived macrophages primed with *H. pylori* towards *E. coli* (**B**): cells primed for 24 h with *H. pylori* (a); cells primed for 24 h with *H. pylori* and restimulated for 5 days with *H. pylori* (b); cells primed for 24 h with *H. pylori* and restimulated for 5 days with *M. bovis* BCG (c); cells primed for 24 h with *H. pylori*, restimulated for 5 days with *M. bovis* BCG and an additional 24 h with *H. pylori* (d). Results are presented as median fluorescence units (RFU) ratio ± range of three independent experiments. The difference statistically significant when *p* < 0.05 in Mann–Whitney *U* test. *Cells stimulated versus unstimulated (according to the time of stimulation). BCG or *H. pylori* MOI 10:1.

It was interesting to know whether the enhanced phagocytic activity of THP-1 monocytes using the reference green labeled *E. coli* particles will last in THP-1 derived macrophages after longer priming of phagocytes with BCG or after restimulation of cells with homologous or heterologous bacterial agent, BCG or *H. pylori*, respectively. Alternatively, whether downregulation of phagocytic activity of macrophages towards fluorescently labeled *E. coli* by *H. pylori* might be reversed by restimulation of cells with BCG. These experimental variants might reflect better the activity of immunocompetent cells exposed to immunomodulating factors during chronic *H. pylori* infection. In this part of the study we used THP-1 derived macrophages, which are long living cells, and evaluated their phagocytic capacity towards fluorescently labeled *E.coli* in Vybrant phagocytosis assay. The viability of control macrophages was compatible with the viability of cells, which were stimulated according to the following schedule: 24 h priming, 24 h priming and 5 days restimulation as well as 24 h priming, 5 days restimulation and 24 h of additional restimulation, was above 90% as showed by the reference MTT reduction assay (data not shown). Cells primed for 24 h with BCG showed significantly increased phagocytic activity towards *E.coli* than control cells in RPMI-1640 medium alone (Fig. 2). The elevated phagocytic activity of macrophages primed with onco-BCG was restored after 5 days restimulation of them with homologic BCG (Fig. 2Aa,b) while diminished after 5 days restimulation with *H. pylori* (Fig. 2A,c). Additional 24 h restimulation of cells with BCG did not result in re-enhancement of phagocytosis (Fig. 2A,d). However, the phagocytic activity of macrophages remained significantly higher than such activity of control cells. By comparison macrophages primed with *H. pylori* for 24 h and then restimulated for 5 days with the same bacteria did not show enhanced ability to engulf *E. coli* (Fig. 2Ba,b). However, the phagocytic activity of cells primed with *H. pylori* was significantly enhanced after restimulation of them for 5 days with BCG, and was restored even after additional 24 h restimulation of cells with *H. pylori* (Fig. 2Bc,d).

The phenomenon of increased phagocytosis of *E. coli* bioparticles by THP-1 derived macrophages exposed to BCG mycobacteria (priming and restimulation) observed by us in vitro might be discussed in the context of cell exposure to *H. pylori*, which modulate negatively a macrophage phagocytic capacity. We showed that the ability of macrophages exposed to BCG-onco to engulf *E.coli* bioparticles was restored even after restimulation of cells with *H. pylori*. Furthermore, very low phagocytic activity of macrophages primed with *H. pylori*, was significantly enhanced after restimulation of cells with BCG. Further in vivo studies are necessary to see whether BCG mycobacteria can effectively activate phagocytes to prevent *H. pylori* infection or to stimulate phagocytes towards *H. pylori* during ongoing infection. In parallel study using a model of *Caviae porcellus* we showed that inoculation of animals with BCG prior to infection with *H. pylori* resulted in diminished amount of mucin receptors to *H. pylori* adhesins, and thus lower colonization of gastric mucosa with these bacteria was observed^36^. Mycobacteria are relatively intracellular pathogens that through numerous cell to cell interactions promote their own uptake by phagocytes^37^. On the other hand, some *H. pylori* adhesins through strong binding to phagocytes may inhibit their ability to engulf *H. pylori*^13^. It is possible that mycobacteria, thanks to their own broad interactions with phagocytes, may enhance phagocytic properties of these cells towards other microorganisms, including *H. pylori*. It has been shown that 19 kD antigen of *M. tuberculosis* is a major adhesin that binds the mannose receptor of THP-1 monocytes and promotes phagocytosis of mycobacteria^38^. These lectin-like interaction of mycobacteria with monocytes/macrophages potentially may interfere with an interactions of *H. pylori* in driving phagocytosis.

### Enhanced expression of cell surface integrins CD11b, CD11d, CD18 in response to priming and restimulation of macrophages with *M. bovis* BCG

**Figure 3:**
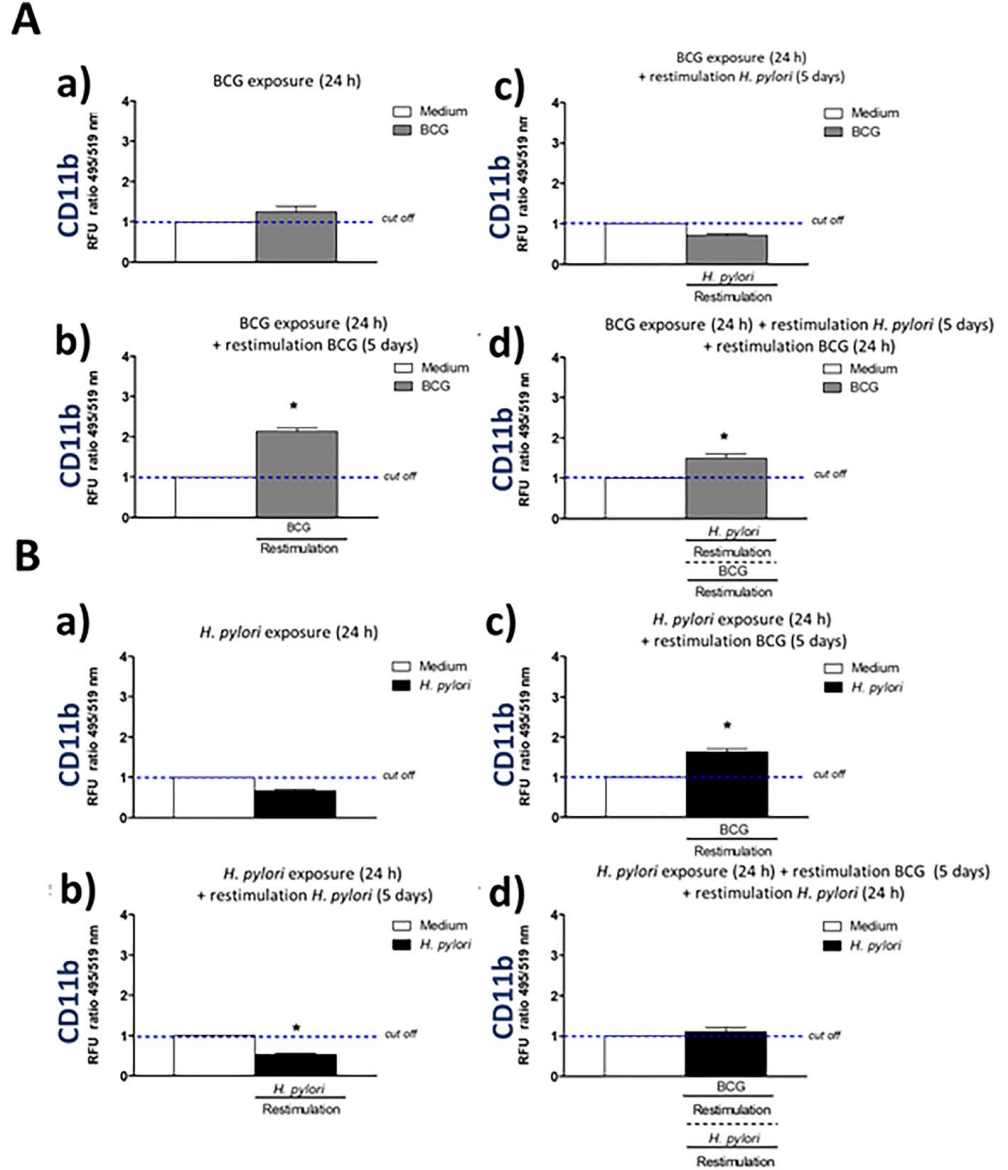
Positive modulation of CD11b expression in THP-1 macrophages by *M. bovis* BCG. Cells were primed with *M. bovis* BCG or *H. pylori* and then they underwent restimulation with homologous or heterologous microbial agent. THP-1 macrophages primed with *M. bovis* BCG (**A**): cells primed for 24 h with *M. bovis* BCG (a); cells primed for 24 h with *M. bovis* BCG and restimulated for 5 days with *M. bovis* BCG (b); cells primed for 24 h with *M. bovis* BCG and restimulated for 5 days with *H. pylori* (c); cells primed for 24 h with *M. bovis* BCG then restimulated for 5 days with *H. pylori* and for an additional 24 h with *M. bovis* BCG (d). THP-1 macrophages primed with *H. pylori* (**B**): cells primed for 24 h with *H. pylori* (a); cells primed for 24 h with *H. pylori* and restimulated for 5 days with *H. pylori* (b); cells primed for 24 h with *H. pylori* and restimulated for 5 days with *M. bovis* BCG (c); cells primed for 24 h with *H. pylori*, restimulated for 5 days with *M. bovis* BCG and an additional 24 h with *H. pylori* (d). The above variants of cells were stained using the fluorescently labeled specific anti-CD11b antibodies. Results are presented as median fluorescence units (RFU) ratio ± range of three independent experiments. The difference statistically significant when *p* < 0.05 in Mann–Whitney *U*. *Cells stimulated versus unstimulated (according to the time of stimulation). BCG or *H. pylori* MOI 10:1.

**Figure 4:**
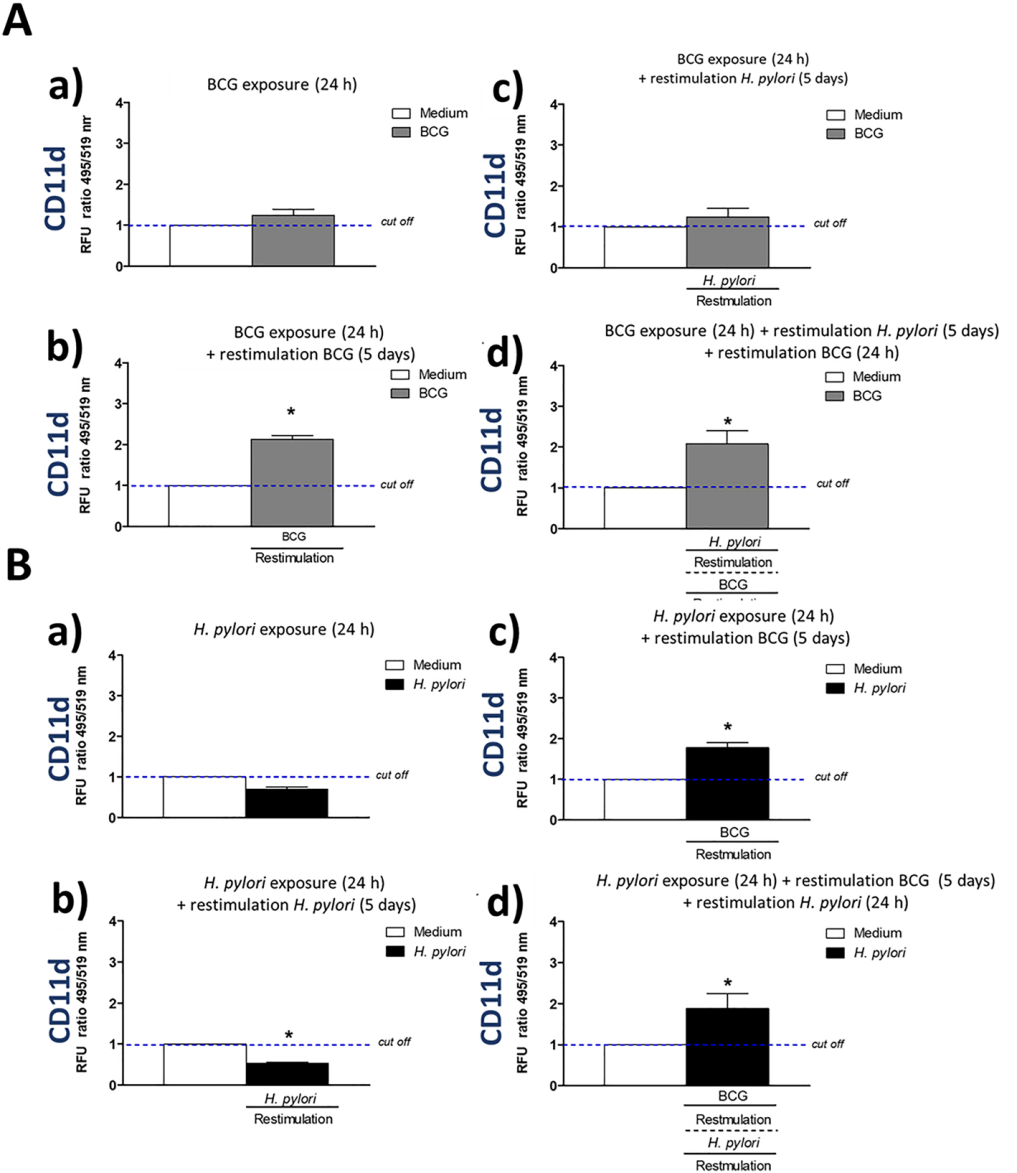
Positive modulation of CD11d expression in THP-1 macrophages by *M. bovis* BCG. Cells were primed with *M. bovis* BCG or *H. pylori* and then underwent restimulation with homologous or heterologous microbial agent. THP-1 macrophages primed with *M. bovis* BCG (**A**): cells primed for 24 h with *M. bovis* BCG (a); cells primed for 24 h with *M. bovis* BCG and restimulated for 5 days with *M. bovis* BCG (b); cells primed for 24 h with *M. bovis* BCG and restimulated for 5 days with *H. pylori* (c); cells primed for 24 h with *M. bovis* BCG then restimulated for 5 days with *H. pylori* and for an additional 24 h with *M. bovis* BCG (d). THP-1 macrophages primed with *H. pylori* (**B**): cells primed for 24 h with *H. pylori* (a); cells primed for 24 h with *H. pylori* and restimulated for 5 days with *H. pylori* (b); cells primed for 24 h with *H. pylori* and restimulated for 5 days with *M. bovis* BCG (c); cells primed for 24 h with *H. pylori*, restimulated for 5 days with *M. bovis* BCG and an additional 24 h with *H. pylori* (d). The above variants of cells were stained using the fluorescently labeled specific anti-CD11d antibodies. Results are presented as median fluorescence units (RFU) ratio ± range of three independent experiments. The difference statistically significant when *p* < 0.05 in Mann–Whitney *U* test. *Cells stimulated versus unstimulated (according to the time of stimulation). BCG or *H. pylori* MOI 10:1.

**Figure 5:**
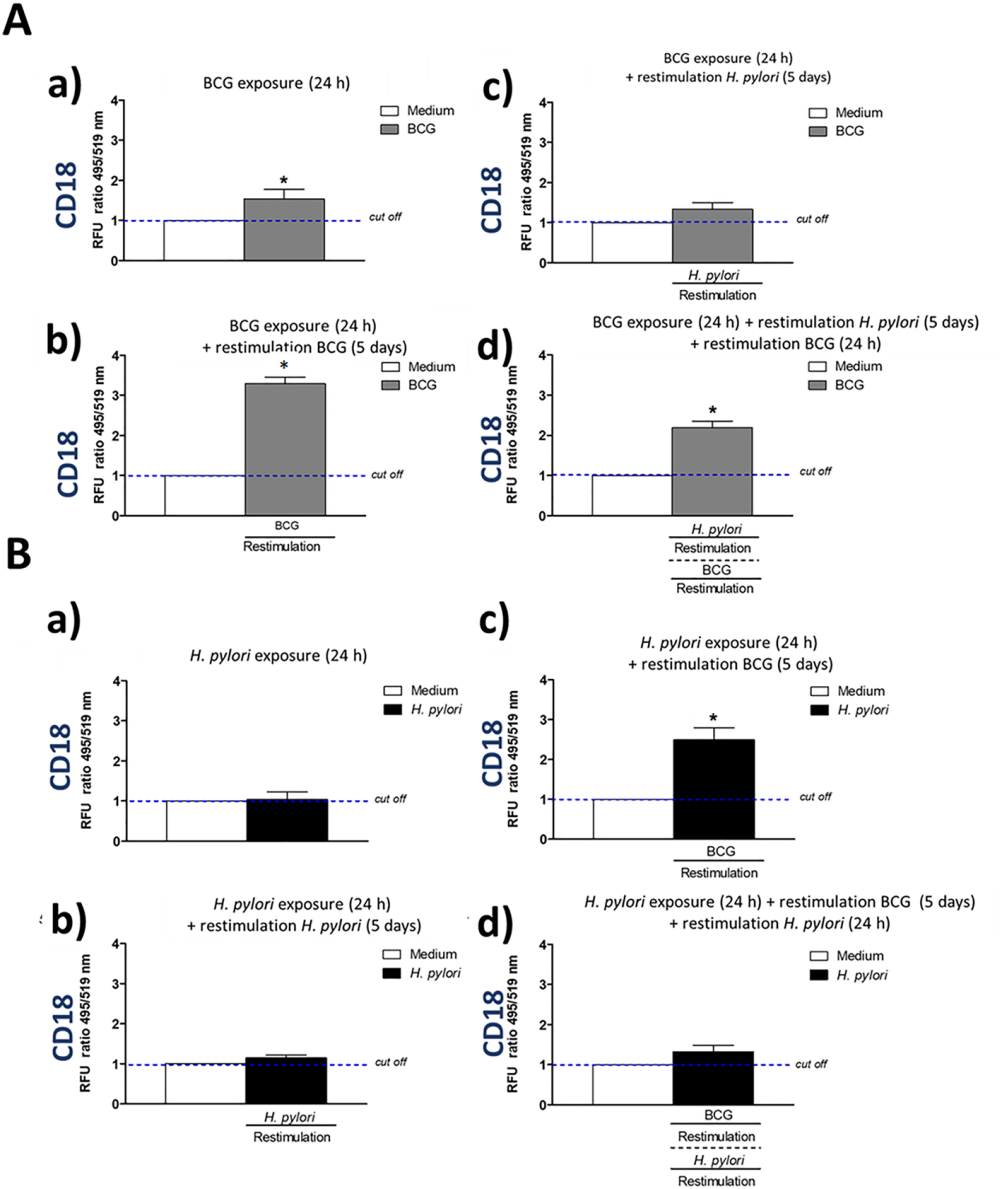
Positive modulation of CD18 expression in THP-1 macrophages by *M. bovis* BCG. Cells were primed with *M. bovis* BCG or *H. pylori* and then underwent restimulation with homologous or heterologous microbial agent. THP-1 cells primed with *M. bovis* BCG (**A**): cells primed for 24 h with *M. bovis* BCG (a); cells primed for 24 h with *M. bovis* BCG and restimulated for 5 days with *M. bovis* BCG (b); cells primed for 24 h with *M. bovis* BCG and restimulated for 5 days with *H. pylori* (c); cells primed for 24 h with *M. bovis* BCG then restimulated for 5 days with *H. pylori* and for an additional 24 h with *M. bovis* BCG (d). THP-1 macrophages primed with *H. pylori* (**B**): cells primed for 24 h with *H. pylori* (a); cells primed for 24 h with *H. pylori* and restimulated for 5 days with *H. pylori* (b); cells primed for 24 h with *H. pylori* and restimulated for 5 days with *M. bovis* BCG (c); cells primed for 24 h with *H. pylori*, restimulated for 5 days with *M. bovis* BCG and an additional 24 h with *H. pylori* (d). The above variants of cells were stained using the fluorescently labeled specific anti-CD18 antibodies. Results are presented as median fluorescence units (RFU) ratio ± range of three independent experiments. The difference statistically significant when *p* < 0.05 in Mann–Whitney *U* test. BCG or *H. pylori* MOI 10:1.

We wanted to explain a potential background of increasing by BCG the phagocytic activity of THP-1 derived macrophages towards *E. coli*, which was showed in this study. The role of interactions between mycobacteria and macrophage receptors in increasing a phagocytic properties of these cells has been suggested^34^. In this study we assessed a deposition on phagocytes of selected surface molecules CD11b, CD11d, CD18 (Figs. 3, 4, 5) mediating an interaction of monocytes/macrophages with vascular endothelium in vivo and involved in pathogen recognition and phagocytosis, including complement dependent phagocytosis, as well as in cell survival and T cell tolerance^39–44^. In our experimental model, BCG mycobacteria induced an increased deposition of the above surface integrins on THP-1 macrophages (Figs. 3Aa,b, 4Aa,b, 5Aa,b). This deposition was diminished during restimulation of cells for 5 days with *H. pylori* (Figs. 3Ab,c, 4Ab,c, 5Ab,c), however, the exposure of cells to BCG for additional 24 h resulted in upregulation of studied surface molecules (Figs. 3Ad, 4Ad, 5Ad). These results suggest that monocytes/macrophages primed and restimulated with BCG are able to respond by an enhanced expression of CD11b, CD11d, CD18, despite its temporary inhibition due to cell exposure to *H. pylori.* The sensitivity of BCG treated cells to *H. pylori* modulation may indicate that BCG-driven effects might be not stable suggesting the lack of an innate memory phenomenon^25^. However, it is interesting, that BCG mycobacteria were able to upregulate the expression of CD11b, CD11d, and CD18 in THP-1 macrophages primed with *H. pylori* (Figs. 3Bc, 4Bc, 5Bc). *H. pylori* bacteria did not enhance the expression of studied integrins or even decreased their deposition on phagocytes (Figs. 3Ba,b, 4Ba,b, 5Ba,b). In the case of CD11d, an increased expression, was observed even on cells that were subjected to an additional 24 h restimulation with *H. pylori*, started after 5 day restimulation of cells with BCG (Fig. 4Bd), but not for CD11b, CD18 (Figs. 3Bd, 5Bd).

These observations suggest that BCG mycobacteria are able to induce the expression of the studied functional surface molecules in monocytes/macrophages even in cells exposed to negative modulator like *H. pylori*.

*H. pylori* and soluble components of these bacteria, including LPS, induce an elevated oxidative stress in the gastric mucosa in conjunction with an increased epithelial cell apoptosis^7,33^. Ganguly et al.^44^ showed that *M. tuberculosis* secretory proteins CFP-10, ESAT-6 and the CFP10:ESAT6 complex may inhibit LPS-induced nuclear factor (NF)-kappa B transactivation by downregulation of reactive oxygen species production. It has been found that bacterial LPS specifically inhibited mouse macrophage uptake of apoptotic neutrophils through suppression of anti-inflammatory growth arrest-specific gene 6 (Gas6), and induction of tumor necrosis factor (TNF)-α^45^. The positively modulated expression of CD11b, CD11d, and CD18 on THP-1 macrophages by BCG-onco showed in this in vitro study may suggest that such upregulation is also possible in vivo. In the further study this hypothesis will be verified in vivo on an experimental model of *H. pylori* infection in *Caviae porcellus*, which was characterized by us in terms of inflammation and an immune responses^46^.

### Upregulation by *M. bovis* BCG of LPS receptors CD14/sCD14 and sMCP

During infection, the immune cells mainly monocytes and macrophages respond to bacterial LPS, present in tissues or the bloodstream, via the Toll-like receptor 4 (TLR-4)/CD14 signaling in conjunction with soluble CD14 (sCD14) and lipopolysaccharide binding protein (LBP). These interactions trigger pro-inflammatory reactions facilitating eradication of the invading bacteria^47^. It has been shown that an experimental exclusion of TLR4-dependent cell signaling during low-grade polymicrobial sepsis resulted in an impaired bacterial clearance and thereby worsened organ injury leading to a higher mortality of mice^48,49^. On the other hand, an exaggerated and uncontrolled pro-inflammatory signaling triggered by TLR4/CD14 during infection can lead to sepsis^50^. As different forms of endotoxin are present in many micro-organisms, the response of the host to non-*E. coli* LPS may be weaker as compared to *E. coli*-derived endotoxin. Thus, other bacterial infections may require the full inflammatory potential of the endotoxin response. The phosphorylation and acylation of *H. pylori* lipid A, a part of LPS, is lower as compared to typical LPS of gut pathogens like *E. coli*^51^. In the previous study we showed that phagocytic activity of human granulocytes towards *H. pylori* was diminished in the milieu of LPS of these bacteria^16^, and other components of these bacteria like surface haemagglutinins and heparin binding proteins may be involved in an inhibition of *H. pylori* engulfment^13^. Also intracellular survival of these bacteria can be affected due to closing of *H. pylori* in cytoplasmic megasomes^15^. LPS *H. pylori* also induced apoptosis of macrophages diminishing their activity as antigen presenting cells^52^. In this study we asked whether *M. bovis* BCG may increase the expression of membrane CD14 and the secretion of sCD14 (Supplementary Figs. S1, S2).

The expression of CD14 on THP-1 macrophages exposed for 24 h to BCG was similar as in control cells sub-cultured in medium alone (Supplementary Fig. S1Aa). Restimulation of such cells for 5 days with homologous BCG, but not with heterologous agent as *H. pylori*, resulted in significantly increased expression of CD14 (Supplementary Fig. S1Ab,c). Furthermore, the priming of THP-1 derived macrophages for 24 h with BCG mycobacteria resulted in an enhancement CD14 membrane receptor expression on cells, which were then restimulated for 5 days with *H. pylori* (Supplementary Fig. S1Ac,d). Priming of phagocytes with *H. pylori* and then restimulation them with these homologous bacteria did not upregulate CD14 expression (Supplementary Fig. S1Ba,b). Deposition of CD14 was significantly increased only on cells primed with *H. pylori* and restimulated for 5 days with BCG (Fig. S1Bc). However, additional restimulation of such cells with *H. pylori* resulted in lower CD14 deposition (Supplementary Fig. S1Bc,d). These results again suggest that BCG mycobacteria and *H. pylori* drive different effects in THP-1 macrophages, increasing or diminishing a CD14 expression, respectively.

Also secretion of sCD14 was significantly increased in cell culture supernatants containingTHP-1 cells primed and restimulated with BCG, and even after restimulation of cells with *H. pylori* (Fig. S2). It remained higher than in control cells, which were sub-cultured in medium alone (Supplementary Fig. S2a–d). Priming and restimulation of macrophages with *H. pylori* alone did not stimulate these cells to sCD14 secretion (Supplementary Fig. S2Ba,b). However, BCG restimulation of cells primed with *H. pylori*, which did not release sCD14, resulted in an increased production of sCD14 (Supplementary Fig. S2Bc), which remained enhanced after an additional 24 h exposure of cells to *H. pylori* (Supplementary Fig. S2Bd). Increased secretion by macrophages stimulated with BCG a soluble CD14 receptor for bacterial LPS and an increased membrane CD14 expression, may reflect a higher potential of these cells to respond to infectious agents, via molecular pattern such as LPS.

In the inflammatory milieu, phagocytes are recruited via endogenous mediators such as soluble macrophage protein 1 (sMCP-1). In our experimental in vitro model only THP-1 cells primed or primed and restimulated with BCG delivered sMCP-1, and the effect of cells exposed to BCG twice was higher than the effect of cells exposed only once (Supplementary Fig. S3Aa,b). *H. pylori* downregulated sMCP-1 production by THP-1 macrophages exposed to BCG, however an additional 24 h stimulation of such cells with mycobacteria resulted in an increased sMCP-1 secretion (Supplementary Fig. S3Ac,d). By comparison, in cultures of THP-1 cells, which were primed or primed and restimulated with *H. pylori* the sMCP-1 concentration was on the level of control culture (cells in medium alone), and did not change after restimulation of cells with BCG (Supplementary Fig. S3). These results indicate that macrophages treated with BCG delivered sMCP-1, which in vivo potentially may increase the local population of monocytes/macrophages. On the contrary, cells primed or primed and restimulated with *H. pylori* did not respond by sMCP-1 production, even after restimulation with BCG. These may suggest that only in cells exposed to BCG, but not to *H. pylori* this activity can be induced, and restored.

### Increased global monocyte DNA methylation induced by *M. bovis* BCG

**Figure 6:**
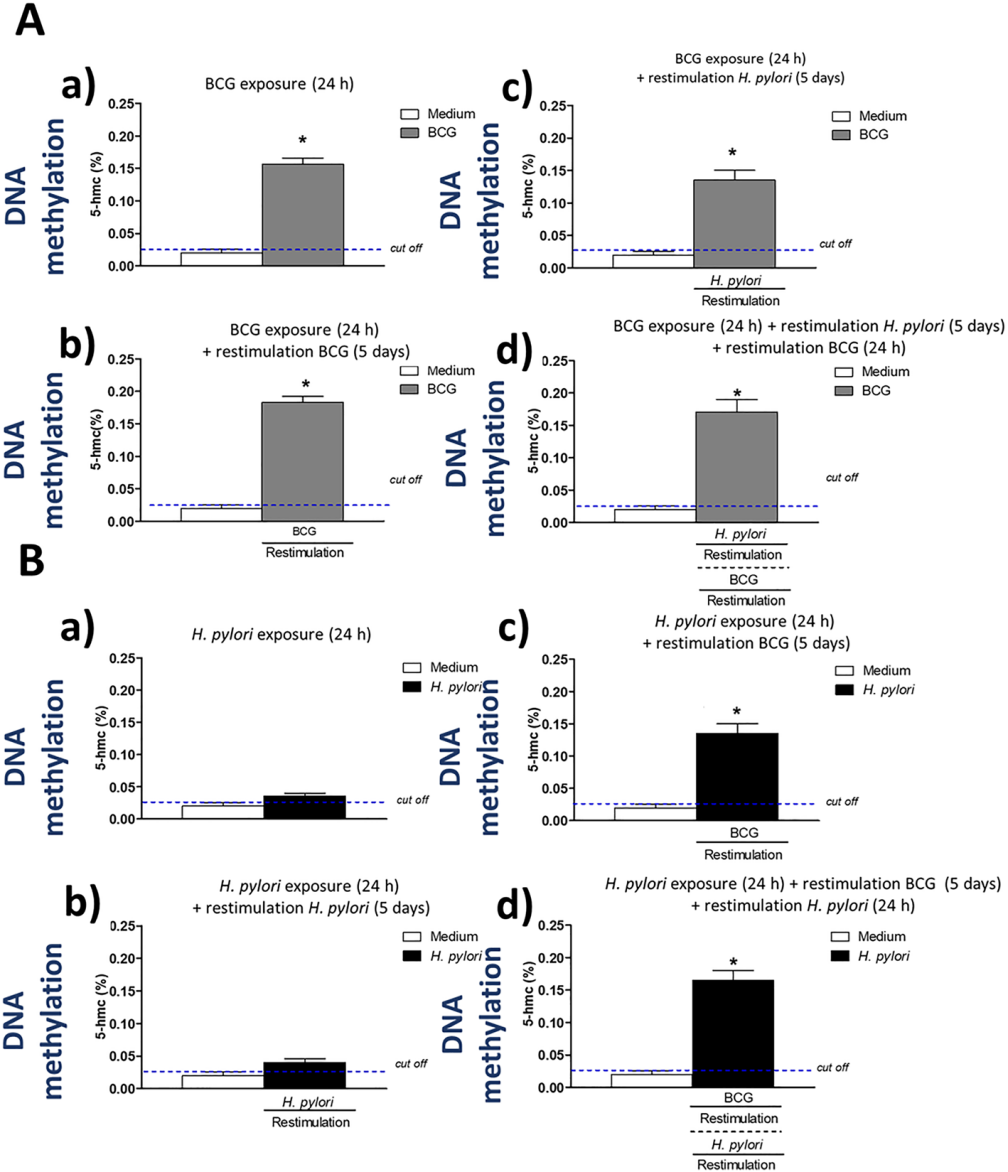
Global DNA methylation. Cells were primed with *M. bovis* BCG or *H. pylori* and then underwent restimulation with homologous or heterologous microbial agent. THP-1 macrophages primed with *M. bovis* BCG (**A**): cells primed for 24 h with *M. bovis* BCG (a); cells primed for 24 h with *M. bovis* BCG and restimulated for 5 days with *M. bovis* BCG (b); cells primed for 24 h with *M. bovis* BCG and restimulated for 5 days with *H. pylori* (c); cells primed for 24 h with *M. bovis* BCG then restimulated for 5 days with *H. pylori* and for an additional 24 h with *M. bovis* BCG (d). THP-1 macrophages primed with *H. pylori* (**B**): cells primed for 24 h with *H. pylori* (a); cells primed for 24 h with *H. pylori* and restimulated for 5 days with *H. pylori* (b); cells primed for 24 h with *H. pylori* and restimulated for 5 days with *M. bovis* BCG (c); cells primed for 24 h with *H. pylori*, restimulated for 5 days with *M. bovis* BCG and an additional 24 h with *H. pylori* (d). The global DNA methylation was determined by the ELISA with high DNA affinity strip wells, and capture as well as detection antibodies specific for 5 hmC were used. The quantity of hydroxymethylated DNA fragments was detected colorimetrically at OD = 450 nm. The percentage of 5hmC in DNA samples was calculated in reference to standard curve. Results are presented as median ratio ± range of three independent experiments. The difference statistically significant when *p* < 0.05 in Mann–Whitney *U* test. *Cells stimulated versus unstimulated (according to the time of stimulation). BCG or *H. pylori* MOI 10:1.

An increased DNA methylation, is a proposed marker of an increased memory-like monocyte/macrophage activity without changing the DNA sequence^53–56^. In this study we asked whether in THP-1 derived macrophages primed or primed and restimulated with BCG-onco the global DNA methylation was increased (Fig. 6). We showed that priming of THP-1 cells and each variant of restimulation of these cells with *M. bovis* BCG resulted in an increased global DNA methylation (Fig. 6Aa–d, Ba–d).

## Conclusion

Using in vitro model of human THP-1 monocytes or THP-1 derived macrophages, we focused on searching whether *M. bovis* onco-BCG may increase the phagocytic activity of monocytes/macrophages towards the reference *E.coli* bioparticles. Alternatively, whether the activity of phagocytes diminished in response to *H. pylori* can be upregulated by BCG. The obtained results indicate that BCG-onco mycobacteria improved the phagocytic activity of THP-1 monocytes and THP-1 derived macrophages towards *E.coli*. Furthermore, the BCG priming and restimulation of THP-1 macrophages resulted in an increased expression of several functional cell surface molecules and secretion of macrophage chemotactic protein. In THP-1 cells primed or primed and restimulated with BCG the global DNA methylation was enhanced as compared to cells, which were not exposed to BCG. This may indicate that BCG induce in monocytes/macrophages epigenetic changes. Our preliminary results also indicate that THP-1 monocytes exposed to *H. pylori* were not able to engulf these bacteria while when primed with BCG mycobacteria showed such activity. These interesting result must be confirmed in further study using the quenching methodology of extracellularly attached *H. pylori*. The potential mechanisms of BCG-induced phagocytosis of *H. pylori* require further understanding. These in vitro results prompt further study on in vivo model of *Caviae porcellus* with experimental *H. pylori* infection, which was well characterized in terms of inflammatory and immune responses^46^. Using this model it will be possible to determine whether inoculation of animals with BCG before *H. pylori* infection or during ongoing infection with these bacteria will prevent or diminish colonization, respectively. Our preliminary results on *Caviae porcellus* model showed that inoculation of animals *per os* with onco-BCG resulted in diminished colonization of gastric mucosa with *H. pylori*, which was related to diminished amount of mucin containing receptors for *H. pylori* adhesins^36^. The current in vitro study showed that BCG may enhance phagocytic capacity of monocytes/macrophages which was affected by *H. pylori* and the expression of an important activity markers of macrophages.

## Material and methods

### Cell cultures

Human THP-1 monocytes (purchased in American Type Culture Collection, ATCC TIB-202, Manassas, VA, USA) (TIB 202). Cells were grown in RPMI (Roswell Park Memorial Institute) -1640 medium supplemented with 10% heat-inactivated fetal calf serum (FCS), 100 U/mL penicillin, 100 U/mL streptomycin, 2 mM/ml L-glutamine at 37 °C, (all in Biowest, Nuaillé, France), in an atmosphere of cell culture incubator, containing 5% CO_2_. Cells were passaged every 3 days to maintain cell density < 2 × 10^6^ cells/mL.

### Stimulation of THP-1 derived macrophages

In several experiments human monocytes THP-1 were differentiated into macrophages with 50 nM phorbol-12-myristate-13-acetate (PMA; Sigma-Aldrich, Saint Louis, USA) for 72 h at 37 °C in the condition of cell culture incubator. After 72 h, the adherent macrophages were exposed to the Bacillus Calmette–Guérin (BCG)-onco vaccine (onco-BCG, Biomed, Lublin, Poland) containing *Mycobacterium bovis* bacilli (*M. bovis* BCG) or to *Helicobacter pylori* CCUG 17874 (purchased from Culture Collection Univeristy of Gotehnburg, Sweden). Different time schedules of macrophage priming or restimulation were used to determine the immunomodulatory activity of microbial formulations. In this study we used the procedure described by Bekkering et al.^57^, in own modification: exposure for 24 h to BCG or *H. pylori*; exposure for 24 h to BCG or *H. pylori* and then restimulation for 5 days with homological or heterological microbial agent (BCG/*H. pylori*); exposure for 24 h to BCG or *H. pylori*, restimulation for 5 days with homological or heterological bacterial agent (*H. pylori*/BCG); and additional restimulation for 24 h (BCG/*H. pylori*). The viability of control macrophages compatible with cells, which were stimulated according to the following schedule: 24 h priming, 24 h priming and 5 days restimulation as well as 24 h priming, 5 days restimulation and 24 h of additional restimulation, was evaluated in the reference 3-(4,5-dimethylthiazol-2-yl)-2,5-diphenyltetrazolium bromide (MTT) reduction assay according to ISO norm as previously described^58^.

*M. bovis* BCG bacilli were suspended in RPMI-1640 culture medium to the density of 5 × 10^7^ CFU (colony-forming unit)/mL; *H. pylori* were grown for 72 h on commercial blood agar plates dedicated to these bacteria (Becton Dickinson East Rutherford, New Jersey, USA), in microaerophilic conditions, suspended in RPMI-1640 to the density of 5 × 10^7^ CFU/mL. Both *M. bovis* and *H. pylori* were used for stimulation of THP-1 cells as multiplicity of infection (MOI) of 10:1. Lipopolysaccharide (LPS) *Escherichia coli* (O55:B5 serotype) (Merck Millipore, Burlington, USA), at the concentration 1 µg/mL was used as positive control. After cell priming or priming and restimulation the cells were used in phagocytosis assay, and to assess selected cell surface integrins/receptors and DNA global methylation while cell culture supernatants were used to determine the concentration of selected soluble mediators.

### Phagocytosis

The suspension of THP-1 monocytes in RPMI-1640 culture medium (5 × 10^6^ cells/mL) was applied to the wells of 96-well plate (100 µL/well), and stimulated with live BCG and/or *H. pylori* at MOI of 10:1 in the following variants: fluorescently labeled *H. pylori* 15 or 30 min, fluorescently labeled BCG 15 or 30 min, first unlabeled BCG 15 min and next fluorescently labeled *H. pylori* 15 or 30 min, or simultaneously with fluorescently labeled *H. pylori* and unlabeled BCG mix 15 or 30 min. *H. pylori* rods and onco-BCG mycobacteria were stained with the commercial LIVE/DEAD BacLight (ThermoFisher, Waltham, USA) tracer for 30 min at room temperature^33^. After labeling, the bacteria were centrifuged 2 times to wash away excess dye. The pre-labeled fluorescent bacteria were plated into the wells containing the phagocytes and stimulated as described above. At selected time points, cell culture supernatants were collected and the fluorescence was measured using a multifunctional reader SpectraMax i3 (Molecular Devicesat, San Jose, CA, USA). Cytospin preparations were made from the remaining cells (cells were collected with medium and 300 µl of cell suspension was centrifuged at 300 × g for 10 min). Cells were fixed with 4% formaldehyde, for 20 min, at room temperature, washed 3 times with phosphate buffered saline (PBS) and stained with DAPI fluorochrome (Sigma-Aldrich, Saint Louis, USA) (1μ DAPI/1000 μL PBS), for 15 min under the conditions as above. Cells were imaged under confocal microscopy (Leica TCS SPE, Wetzlar, Germany) at the wavelength for each fluorochrome: fluorescein isothiocyanate (FITC)/ BacLight (excitation 495 nm, emission 519 nm), DAPI (excitation 345 nm, emission 455 nm) at a magnification of 63 × .

Phagocytic activity of THP-1 cells was also assessed using fluorescently labeled *Escherichia coli* bioparticles from Vybrant Phagocytosis Assay Kit (ThermoFisher Scientific, Waltham, USA), as recommended by the manufacturer. For this purpose the various variants of cells were used: stimulated with *H. pylori* 15 or 30 min, stimulated with BCG 15 or 30 min, exposed first to BCG 15 min and next to *H. pylori* 15 or 30 min, or exposed simultaneously to *H. pylori* and BCG mix 15 or 30 min. To see how longer and repeated exposure of cells to BCG or *H. pylori* will influence the phagocytic activity of THP-1 derived macrophages we used different variants of cells, which were primed with BCG or *H. pylori* for 24 h, and then restimulated for 5 days or additional 24 h with homologous or heterologous bacterial agent (BCG/*H. pylori*). Intensity of phagocytosis was measured by using a multifunctional reader SpectraMax i3 (Molecular Devices, San Jose, CA, USA) reader at 495 nm (excitation) and 515 nm (emission).Three independent experiments were performed in triplicate for each variant.

### Determination of cell surface markers: immunofluorescence

The macrophages were prepared for staining with primary and secondary antibodies fluorescently labeled, as previously described^7^. To assess macrophage surface markers of activation, we used the following monoclonal antibodies: rabbit anti-CD11b, mouse anti-CD11d, rat anti-CD14, rabbit anti-CD18 (all from ThermoFisher Waltham, USA), diluted 1:200 in 1% bovine serum albumin (BSA) in PBS. The macrophages were then treated with the appropriate secondary antibody: goat anti-rabbit, goat anti-mouse or goat anti-rat labeled with Alexa Fluor488 or Alexa Fluor 568 (Invitrogen, CA USA) diluted 1:200 I 1% BSA/PBS. The intensity of fluorescence was measured by using a multifunctional reader SpectraMax i3 (Molecular Devices, San Jose, CA, USA) at the appropriate wavelengths: for Alexa Fluor488 (excitation 495 nm, emission 519 nm), for Alexa Fluor 568 (excitation 591 nm, emission 608 nm). Three independent experiments were performed in triplicate.

### Determination by the ELISA of soluble mediators delivered by macrophages

Cell culture supernatants were tested for soluble CD14 (sCD14), and macrophage chemotactic protein (MCP)-1 by the commercial Enzyme Linked Immunosorbent Assay—ELISA (ThermoFisher, Waltham, USA), with a sensitivity of 6 pg/mL (sCD14), and 3.3 pg/mL (MCP-1). The ELISA assays dedicated to each mediator were developed as recommended by the manufacturer. Three independent experiments were performed in triplicate.

### Isolation of macrophage DNA and estimation of its global methylation

The suspensions of macrophages, which underwent priming or priming and restimulation as described above were transferred to individual tubes and centrifuged (400 × g for 10 min, at room temperature). Cellular pellets were suspended in 200 μL of TRIS buffer and were used for DNA isolation according to the Genomic Mini DNA purification protocol (A&A Biotechnology, Gdansk, Poland). Briefly, samples were transferred to 2 mL DNAse free tubes, treated with lysis buffer and proteinase K for 20 min, and for 5 min with 5 μL of RNAase A (10 mg/mL). Following incubation (5 min at 70 °C) with TRIS elution buffer, 20 s vortexing and centrifugation (250 × g) the supernatants were transferred to microcolumns. Purified DNA samples were stored at − 20 °C. The efficiency and purity of DNA were verified spectrophotometrically by Nanophotometer Pearl (Implen, Westlake, USA), 100–125 ng/μL, and A 260/280 ratio = 1.6, respectively. DNA Methylation enzyme‐linked immunosorbent assay (ELISA; Epigenetek, Farmingdale, USA), with high DNA affinity strip wells, and capture as well as detection antibodies specific for 5 hmC were used. The quantity of hydroxymethylated DNA fragments was detected colorimetrically at OD = 450 nm. The percentage of 5hmC in DNA samples was calculated in reference to standard curve ranging from 0.02 to 1%. To calculate the percentage of 5 hmC in total DNA the following formula was used: 5‐hmC% = (sample OD − negative control OD/slope × 100 ng) × 100%

### Statistical analysis

Data were expressed as the median ± range. The differences between groups were tested using the non-parametric Mann–Whitney *U* test. For statistical analysis the Statistica 13 PL software (https://statistica.software.informer.com/13.3software (Krakow, Poland), was used. Results were considered statistically significant when *p* < 0.05.

## Supplementary Information

Supplementary Information.

## Data Availability

All data generated or analyzed during this study are included in this published article or supplementary file.
