# Supplementary material for: Mycobacterium bovis BCG increase the selected determinants of monocyte/macrophage activity, which were diminished in response to gastric pathogen Helicobacter pylori
Source: Sci Rep. 2023 Feb 22;13:3107. doi: 10.1038/s41598-023-30250-6 (PMC9944772; doi:10.1038/s41598-023-30250-6)
Supplement: Supplementary file 1 — Supplementary Information. [file 41598_2023_30250_MOESM1_ESM.docx]

***Mycobacterium bovis* BCG increase the selected determinants of monocyte/macrophage activity, which were diminished in response to gastric pathogen *Helicobacter pylori***

Weronika Gonciarz, Maciej Chyb, Magdalena Chmiela


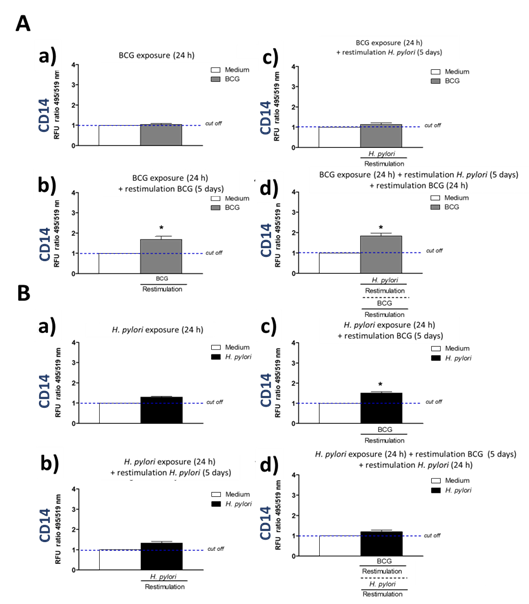


**Supplementary Figure S1.**

Upregulation of CD14 membrane receptor in THP-1 macrophages by *M. bovis* BCG.

Cells were primed with *M. bovis* BCG or *H. pylori* and then underwent restimulation with homologous or heterologous microbial agent. THP-1 macrophages primed with *M. bovis* BCG **(A)**: cells primed for 24h with *M. bovis* BCG **(a)**; cells primed for 24h with *M. bovis* BCG and restimulated for 5 days with *M. bovis* BCG **(b)**; cells primed for 24h with *M. bovis* BCG and restimulated for 5 days with *H. pylori* **(c)**; cells primed for 24h with *M. bovis* BCG then restimulated for 5 days with *H. pylori* and for an additional 24h with *M. bovis* BCG **(d)**. THP-1 macrophages primed with *H. pylori* **(B)**: cells primed for 24h with *H. pylori* **(a)**; cells primed for 24h with *H. pylori* and restimulated for 5 days with *H. pylori* **(b)**; cells primed for 24h with *H. pylori* and restimulated for 5 days with *M. bovis* BCG **(c)**; cells primed for 24h with *H. pylori*, restimulated for 5 days with *M. bovis* BCG and an additional 24h with *H. pylori* **(d)**. The above variants of cells were stained using the fluorescently labeled specific anti-CD14 antibodies. Results are presented as median fluorescence units (RFU) ratio ± range of three independent experiments. The difference statistically significant when *p<0.05* in Mann-Whitney U test. *cells stimulated vs. unstimulated (according to the time of stimulation). BCG or *H. pylori* MOI 10:1.


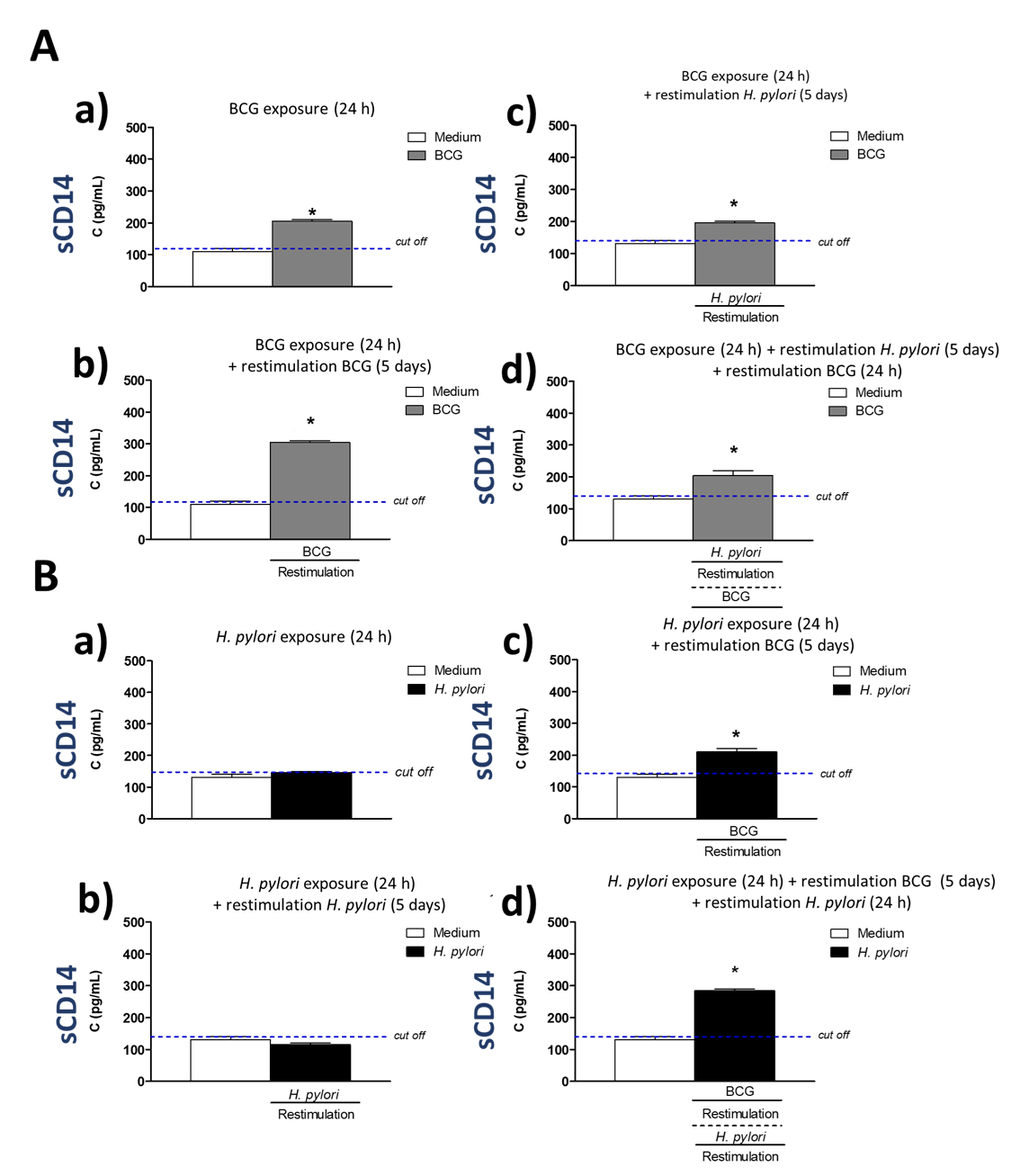


**Supplementary Figure S2**

Enhanced secretion of soluble CD14 (sCD14) by THP-1 macrophages primed or restimulated with *M. bovis* BCG.

Cells were primed with *M. bovis* BCG or *H. pylori* and then underwent restimulation with homologous or heterologous microbial agent. THP-1 macrophages primed with *M. bovis* BCG **(A)**: cells primed for 24h with *M. bovis* BCG **(a)**; cells primed for 24h with *M. bovis* BCG and restimulated for 5 days with *M. bovis* BCG **(b)**; cells primed for 24h with *M. bovis* BCG and restimulated for 5 days with *H. pylori* **(c)**; cells primed for 24h with *M. bovis* BCG then restimulated for 5 days with *H. pylori* and for an additional 24h with *M. bovis* BCG **(d)**. THP-1 macrophages primed with *H. pylori* **(B)**: cells primed for 24h with *H. pylori* **(a)**; cells primed for 24h with *H. pylori* and restimulated for 5 days with *H. pylori* **(b)**; cells primed for 24h with *H. pylori* and restimulated for 5 days with *M. bovis* BCG **(c)**; cells primed for 24h with *H. pylori*, restimulated for 5 days with *M. bovis* BCG and an additional 24h with *H. pylori* **(d)**. The sCD14 concentration in cell culture supernatants was determined using the ELISA. Results are presented as median ± range of three independent experiments. The difference statistically significant when *p<0.05* in Mann-Whitney U test. *cells stimulated vs. unstimulated. BCG or *H. pylori* MOI 10:1.


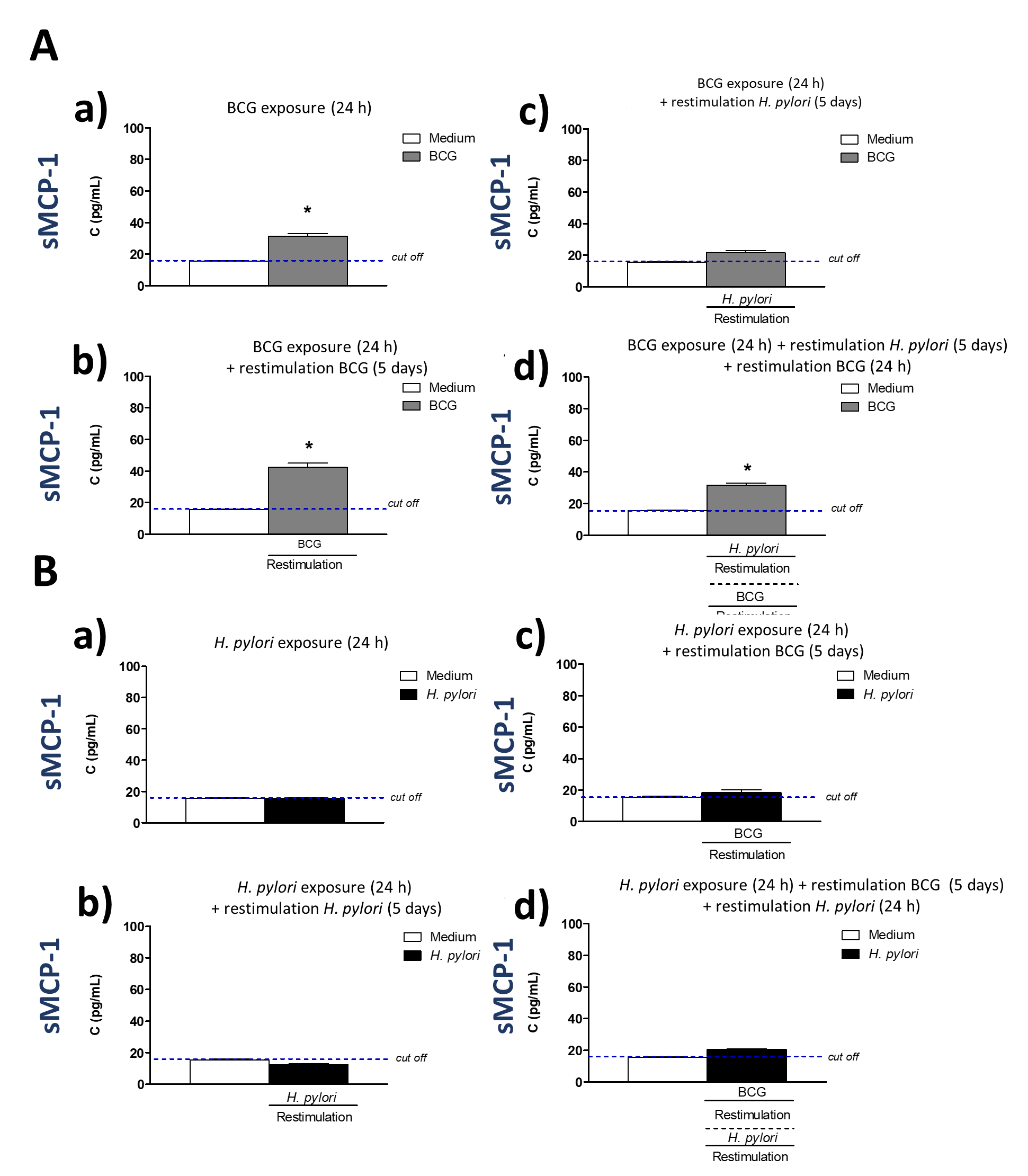


**Supplementary Figure S3.**

**S**ecretion of soluble macrophage chemotactic protein (MCP) – 1 by THP-1 macrophages exposed to BCG mycobacteria and/or *H. pylori*.

Cells were primed with *M. bovis* BCG or *H. pylori* and then underwent restimulation with homologous or heterologous microbial agent. THP-1 macrophages primed with *M. bovis* BCG **(A)**: cells primed for 24h with *M. bovis* BCG **(a)**; cells primed for 24h with *M. bovis* BCG and restimulated for 5 days with *M. bovis* BCG **(b)**; cells primed for 24h with *M. bovis* BCG and restimulated for 5 days with *H. pylori* **(c)**; cells primed for 24h with *M. bovis* BCG then restimulated for 5 days with *H. pylori* and for an additional 24h with *M. bovis* BCG **(d)**. THP-1 macrophages primed with *H. pylori* **(B)**: cells primed for 24h with *H. pylori* **(a)**; cells primed for 24h with *H. pylori* and restimulated for 5 days with *H. pylori* **(b)**; cells primed for 24h with *H. pylori* and restimulated for 5 days with *M. bovis* BCG **(c)**; cells primed for 24h with *H. pylori*, restimulated for 5 days with *M. bovis* BCG and an additional 24h with *H. pylori* **(d)**. The sMCP-1 concentration in cell culture supernatants was determined using the ELISA. Results are presented as median ± range of three independent experiments. The difference statistically significant when *p<0.05* in Mann-Whitney U test. *cells stimulated vs. unstimulated (according to the time of stimulation). BCG or *H. pylori* MOI 10:1.
